# Pursuit of the ideal antiseptic irrigation solution in the management of periprosthetic joint infections

**DOI:** 10.5194/jbji-6-189-2021

**Published:** 2021-05-26

**Authors:** Ahmed Siddiqi, Zuhdi E. Abdo, Bryan D. Springer, Antonia F. Chen

**Affiliations:** 1 Orthopaedic Institute of Central Jersey, a division of Ortho Alliance NJ, 2315 Route 34 South Manasquan, NJ 08736, USA; 2 Hackensack Meridian School of Medicine, Department of Orthopedic Surgery, Hackensack, NJ, USA; 3 Jersey Shore University Medical Center, Department of Orthopedic Surgery, Neptune, NJ, USA; 4 Rutgers New Jersey Medical School, Department of Orthopedics, Newark, NJ, 07103, USA; 5 OrthoCarolina Hip and Knee Center, Department of Orthopedics Atrium Musculoskeletal Institute, Charlotte, NC, 28207, USA; 6 Brigham & Women's Hospital, Department of Orthopedics, Boston, MA, 02115, USA

## Abstract

Irrigation and debridement in the treatment of periprosthetic joint infection (PJI) serve an integral role in the eradication of bacterial burden and subsequent re-infection rates. Identifying the optimal irrigation agent, however, remains challenging, as there is limited data on superiority. Direct comparison of different irrigation solutions remains difficult because of variability in treatment protocols. While basic science studies assist in the selection of irrigation fluids, in vitro results do not directly translate into clinical significance once implemented in vivo. Dilute povidone iodine, hydrogen peroxide, chlorhexidine gluconate, acetic acid, sodium hypochlorite, hypochlorous acid, and preformed combination solutions all have potential against a broad spectrum of PJI pathogens with their own unique advantages and disadvantages. Future clinical studies are needed to identify ideal irrigation solutions with optimal bactericidal properties and low cytotoxicity for PJI treatment.

## 1 Introduction

Periprosthetic joint infection (PJI) and surgical site infection (SSI) remain common and devastating complications after total joint arthroplasty (TJA) (Sloan et al., 2018). Although two-stage revision arthroplasty is considered the gold standard treatment for PJI, debridement, antibiotics, and implant retention (DAIR) as well as one-stage revision arthroplasty may be considered in certain situations in effort to decrease morbidity and mortality associated with additional surgical procedures. However, thorough irrigation and debridement of infectious tissue remains the most critical portion of any of the surgical options for acute and chronic PJI.

The use of multiple additives with normal saline solution irrigation to improve bacterial bioburden eradication is increasing. The optimal irrigation solution should have minimal toxicity while maintaining bactericidal and fungicidal activity at its minimum biofilm eradication concentration (MBEC), which is defined as the lowest concentration needed to diminish the biofilm by 99.9 % (Van Meurs et al., 2014). Additives are divided into three categories: surfactants, antibiotics, and antiseptics. Surfactants contain detergents, such as castile soap or benzalkonium chloride. Antibiotic irrigation most commonly contains bacitracin and/or polymyxin. Antiseptic solutions include povidone iodine, chlorhexidine gluconate (CHG), hydrogen peroxide, sodium hypochlorite, acetic acid, hypochlorous acid, and combination solutions (Table 1). Identifying the ideal irrigation solution remains challenging, however, as there is limited data on superiority.

**Table 1:** Most common antiseptic irrigation solutions.

Antiseptic	MOA	Bacteria	Fungi	Mycobacteria	Spores	Viruses	Biofilm
Povidone iodine	The iodine itself acts as a potent oxidizer to cell membranes and intracellular components, effectively inactivating proteins, nucleotides, and fatty acids in a concentration-dependent manner	Bactericidal	Fungicidal	Mycobactericidal	Sporicidal	Viricidal	Limited effect
Hydrogen peroxide	Produces hydroxyl free radicals that denature proteins, lipids, and deoxyribonucleic acid resulting in cell death.	Bactericidal (gram-positive more than gram-negative)	Fungicidal	–	Sporostatic	–	Limited effect
Chlorhexidine gluconate	CHG binds to negatively charged microbial cell walls, altering the osmotic equilibrium of the cell, and further attacks inner bacterial cytoplasmic membranes or yeast plasma membranes with resultant cytoplasmic clumping.	Bactericidal	–	Mycobacteriostatic	Sporostatic	Viricidal	Some effect
Acetic acid	A weak organic acid that is produced by the oxidation of ethanol. The bactericidal activity of acetic acid hinges on its diffusion through the bacterial cell membrane and lowering pH in a process called ion trapping.	Bactericidal	Fungicidal	Mycobactericidal	–	–	Some effect
Sodium hypochlorite	Produces chloride ions, a potent oxidizer that inhibits protein synthesis and essential lipids in the bacterial cell membrane, resulting in its antimicrobial effect.	Bactericidal	–	–	Sporicidal at higher concentrations	Viricidal	Limited effect
Hypochlorous acid	Created by white blood cells during the oxidative burst to kill pathogens. HOCl is additionally produced by neutrophils, macrophages, monocytes, and myeloperoxidase-catalyzed peroxidation of chloride ions. The residual chloride ions oxidize the surrounding bacterial cells in a similar mechanism to sodium hypochlorite, except in a slightly more acidic pH 5.5.	Bactericidal	Fungicidal	–	Sporicidal at higher concentrations	Viricidal	Limited effect
Bactisure	Physically deconstructs the protective EPS matrix, making pathogens more susceptible to traditional antibiotics and the body's normal defense mechanism. Wound lavage assists with mechanical removal of organisms.	Bactericidal	Fungicidal	–	–	Viricidal	Some effect
Prontosan	Betaine acts as surfactant to aid with debridement. Polyhexanide is a preservative. The combination of the two products provides a lower surface tension than water and improves biofilm removal in wounds.	Bactericidal	Fungicidal	–	–	Viricidal	Some effect

MOA: mechanism of action; EPS: extracellular polymeric substances; HOCl: hypochlorous acid; CHG: Chlorhexidine gluconate.Table content reproduced with permission from Kavolus et al. (2020).

Although high-quality clinical trials exploring surfactant irrigation in PJI management are scarce, the Center for Disease Control (CDC), World Health Organization (WHO), National Institute for Health and Care Excellence (NICE), and 2018 international consensus meeting (ICM) on musculoskeletal infections recommend against the use of antibiotic irrigation in PJI and SSI prevention and treatment (Blom et al., 2019). More recently, the Food and Drug Administration (FDA) has requested withdrawal of bacitracin injections due to high risk of anaphylaxis and complications (FDA, 2020). Furthermore, there are no clear guidelines regarding the optimal irrigation type, amount, or protocol for management of acute or chronic PJI. The purpose of this comprehensive review is to evaluate commercially available antiseptic irrigation solutions and their clinical outcomes and complications for the management of PJI.

## 2 Povidone iodine

Povidone iodine functions as a powerful oxidizer to cell membranes and inactivates intracellular contents in a concentration-dependent method (Ruder and Springer, 2016). Although clinical practice guidelines from the WHO and ICM recommend sterile povidone iodine for the prevention of SSI (Blom et al., 2019; World Health Organization, 2018) there are no guidelines regarding povidone iodine's role in definitive PJI management. The ICM, however, does support the utilization of dilute povidone iodine with a “super majority, strong consensus” during DAIR procedures without further specifying optimal dilution concentrations (Blom et al., 2019). While some studies suggest povidone iodine to be very effective with minimal damage to host tissue at lower concentrations (Rabenberg et al., 2002) other studies report toxicity regardless of concentration (Foresman et al., 1993).

Different povidone-iodine concentrations have been tested against various organisms to determine bactericidal activity relative to host cell viability (Ruder and Springer, 2016). At higher concentrations (20 %), PI has toxicity against human fibroblast cells (Rabenberg et al., 2002). Therefore, dilution is necessary to mitigate PI toxicity to host tissue. However, the optimal PI dilution has yet to be determined. PI is commercially available at 100 g/L (10 %), which is both bactericidal and cytotoxic (Ruder and Springer, 2016; Van Meurs et al., 2014).

### 2.1 Outcomes

A povidone iodine solution (10 %) has been shown to have efficacy against methicillin-sensitive *Staphylococcus aureus* (MSSA) biofilm grown on plastic, cement, and porous titanium with minimal effectiveness of dilute 0.35 % povidone iodine (Premkumar et al., 2020). However, in another in vitro analysis, Goswami et al. (2019) reported 0.3 % povidone iodine to have adequate eradication of MSSA and *Escherichia coli* biofilm with minimal cytotoxicity against human osteoblasts, chondrocytes, and fibroblasts. Other studies have shown povidone irrigation's ability to remove common PJI bacteria biofilm (including MRSA, MSSA, *Staphylococcus epidermidis*, *Haemophilus influenzae*, *Pseudomonas aeruginosa*, and *E. coli*) on orthopaedic materials such as stainless-steel screws, titanium discs, and polyethylene washers (Cichos et al., 2019; Gilotra et al., 2015). There is also no study, to our knowledge, that reports identifiable acquired bacterial resistance or cross-resistance which would make povidone iodine an appealing irrigant adjuvant in PJI treatment.

Recently, Riesgo et al. (2018) compared the utility of dilute povidone-iodine irrigation with vancomycin powder for 20 total hip arthroplasty (THA) and 16 total knee arthroplasty (TKA) PJI cases compared to a matched cohort of patients managed with normal saline without antibiotic powder. The authors reported 83.3 % (30 out of 36) success rate of the povidone-iodine and vancomycin cohort compared to 63.2 % (24 out of 38) success of the control group at 27-month follow-up. Although the re-infection rates were not statistically different between the two groups (0=0.32), the povidone-iodine cohort had a 45 % relative risk reduction and overall DAIR success rate improvement from 63 % to 83.3 %. Currently there is a multicenter, prospective, randomized-controlled trial evaluating vancomycin powder, povidone iodine alone, vancomycin–povidone-iodine combination, and saline in the prevention of PJI. The outcomes of the study may help guide future management of PJI.

### 2.2 Complications

Several studies have raised concerns regarding povidone iodine's cytotoxic effects on human tissue including osteoblasts, myoblasts, chondrocytes, and fibroblasts; however, this in vitro concern has not translated into in vivo studies (Goswami and Austin, 2019a). Povidone iodine has also raised concerns from increasing patient-reported iodine allergies including anaphylaxis (Waran and Munsick, 1995).

Additionally, various factors have been associated with povidone-iodine contamination as multiple organisms, including *Burkholderia cepacia*, *P. aeruginosa*, *Mycobacterium abscessus*, and fungal pathogens, have been reported to be found in aqueous povidone iodine, either directly from the packaged bottles or from dilution with non-sterile saline (Goswami and Austin, 2019b). Recently in October 2020, the Food and Drug Administration (FDA) approved the first sterile commercially available dilute povidone-iodine solution (Surgiphor Wound Irrigation System, Parvizi Surgical Innovation, Philadelphia, PA) for clinical utilization (Surgiphor Wound Irrigation System FDA, 2021). Finally, surgeons who inject liposomal bupivacaine for multimodal analgesia should be cognizant that povidone-iodine lyses liposomes, thereby removing its slow-release effect (Ruder and Springer, 2016). Povidone-iodine irrigation should, therefore, be utilized prior to administration of liposomal bupivacaine.

## 3 Hydrogen peroxide

Hydrogen peroxide produces free radicals that denature proteins, lipids, and deoxyribonucleic acid (DNA) resulting in bacterial and fungal cell death. It is organically present within human tissues and serves diverse roles in the host innate immune response to infection (Lu and Hansen, 2016). It has been proven effective against viruses, bacteria, yeasts, and bacterial spores, with its greatest bactericidal affects against gram-positive organisms (Lu and Hansen, 2016). Hydrogen peroxide's foam further aids in mechanical wound debridement without detrimental effects on the strength of bone–cement interfaces or metal implants, which is critical during acute PJI and DAIR procedures (Lu and Hansen, 2016).

### 3.1 Clinical outcomes

Multiple in vitro studies have shown hydrogen peroxide's ability to reduce a broad spectrum of bacterial biofilm (Glynn et al., 2009; Lu and Hansen, 2016). Glynn et al. (2009) found that different concentrations of hydrogen peroxide (5, 10 mM) inhibited biofilm development by *Staphylococcus epidermidis* compared to an untreated control group. Zubko and Zubko (2013) reported the synergistic effect of hydrogen peroxide and povidone iodine concurrently against 3 bacterial (*S. aureus*, *Pseudomonas aeruginosa*, *E. coli*) and 16 fungal pathogens and found hydrogen peroxide and povidone iodine to be bacteriostatic when used separately and bactericidal when used in conjunction. Synergistic utilization of hydrogen peroxide with other antiseptic solutions has the potential to treat a broader spectrum of pathogens at lower cytotoxic concentrations of each individual solution (Zubko and Zubko, 2013). Further in vivo studies are needed to investigate the role of hydrogen peroxide alone and its synergistic effects with other antiseptic irrigating solutions in PJI management.

### 3.2 Complications

Despite some host cytotoxic effects reported in in vitro investigations, no in vivo studies have shown deleterious effect on host tissues or wound healing. The breakdown of hydrogen peroxide into oxygen gas, however, increases the risk of air embolism, with reports of cardiac arrest in the literature (Henley et al., 2004). Thorough irrigation with normal saline is recommended after hydrogen-peroxide utilization to mitigate such complications.

## 4 Chlorhexidine gluconate

CHG is a cationic bisbiguanide that binds to bacterial and fungal cell walls and alters the intracellular osmotic equilibrium (George et al., 2017). CHG is highly effective against a broad spectrum of pathogens responsible for PJI including MSSA, MRSA, coagulase negative *Staphylococcus* (CNS), gram-negative bacteria, and fungi (George et al., 2017). CHG's bactericidal effect is almost immediate with host tissue contact, with the greatest uptake occurring within 20 s of exposure. Its duration of effect is directly related to both the length of exposure and solution concentration (Weinstein et al., 2008). The FDA recently approved a dilute CHG formulation (0.05 % CHG in sterile water; Irrisept, Innovation Technologies, Inc, Lawrenceville, Georgia) for wound irrigation.

### 4.1 Clinical outcomes

Similar to other antiseptic irrigation efficacy studies, the broad-spectrum effects of CHG have been highlighted in in vitro studies. CHG solutions have been shown to decrease MRSA biofilm load (Schwechter et al., 2011) and *Staphylococcus epidermidis* biofilm (Frisch et al., 2017) on orthopaedic implants using a titanium alloy (Ti-6Al-4V) in vitro models. Clinically, Barros et al. (2019) noted an 89.5 % (34 out of 38) success rate using a CHG scrub brush followed by normal saline irrigation in a DAIR (12 THA, 12 TKA) protocol at 2-year follow-up. Similarly, Byren et al. (2009) used an unknown concentration and volume of CHG irrigation for the treatment of 51 TKA and 52 THA PJIs, noting a success rate of 73.1 % (38 out of 51) and 86.5 % (45 out of 52), respectively. There is no literature, however, to our knowledge, that explores the utility of the commercially available CHG solution alone in the definitive management of PJI. Since the commercially available solution has low CHG concentration (0.05 %), its efficacy against biofilm may be limited based on in vitro studies.

### 4.2 Complications

Although CHG has demonstrated efficacy against gram-positive and gram-negative pathogens (Van Meurs et al., 2014), other studies have shown its antibacterial effect offset by increased host tissue toxicity (Liu et al., 2018). Higher CHG concentrations (0.5 % to 2 %) have been shown to substantially reduce human fibroblast, myoblast, osteoblast, and stromal cell survival (Kavolus et al., 2020; Van Meurs et al., 2014). Furthermore, some studies report antibiotic resistance after CHG exposure, as well as *Enterococcus faecium* and *Pseudomonas* resistance to CHG itself (Kavolus et al., 2020). Hypersensitivity reactions and anaphylaxis, although rare, also remain a concern.

## 5 Acetic acid

Acetic acid, commonly found in vinegar, is a weak organic acid that lowers pH and exerts its bactericidal activity by diffusion through the bacterial cell membrane (Tsang et al., 2018a). It has activity against both gram-positive and gram-negative organisms in both planktonic and biofilm environments (Kavolus et al., 2020; Tsang et al., 2018b). Similar to povidone iodine, CHG, and hydrogen peroxide, orthopaedic implant materials such as stainless steel and titanium are resistant to its corrosive effects (Tsang et al., 2018a).

### 5.1 Clinical outcomes

Acetic acid demonstrates antibiofilm activity against *Pseudomonas aeruginosa* and *S. aureus* with concentrations as low as 0.5 % and 1 %, respectively (Williams et al., 2017). Although acetic acid is noncorrosive to human tissue at concentrations less than 5 % (Hughes and Webber, 2017), Tsang et al. (2018a) reported 5 % acetic acid solution eradicated 96.1 % of MSSA biofilm and a 3 % solution reduced MSSA biofilm by 85.9 %. The MBEC at 10 and 20 min was 15 % and 11 %, respectively (Tsang et al., 2018a).

There is limited in vivo data on acetic acid's effectiveness for treating PJI. Williams et al. (2017) implemented a 20 min soak with 3 % acetic acid during TKA PJI management using DAIR, two-stage exchange arthroplasty and arthrodesis procedures. The authors reported an 87 % (20 out of 23) success rate without any host tissue toxicity resulting in wound complications. Further investigation is warranted to evaluate acetic acid's role against a broad spectrum of common PJI bacterial and fungal pathogens and its safety on surrounding host soft tissue.

### 5.2 Complications

Unlike other antiseptic solutions, 3 % acetic acid requires long soaking times up to 20 min to have optimal concentration and duration against resistant *Pseudomonas* wound infections (Tsang et al., 2018a; Williams et al., 2017). The long duration of acetic acid's intraoperative application must be considered alongside the risks of increased intraoperative times to the patient.

## 6 Sodium hypochlorite

Sodium hypochlorite (NaOCl) commercially sold as Dakin's solution (Century Pharmaceuticals, Inc, Indianapolis, Indiana), is household bleach that has antimicrobial activity against aerobic and anaerobic bacteria, viruses, and fungi at lower concentrations (0.005 %–0.025 %), while sparing host fibroblasts and chondrocytes. Sodium hypochlorite produces chloride ions, a potent oxidizer that inhibits protein synthesis and essential lipids in the bacterial and fungal cell membrane resulting in its bactericidal and fungicidal effect (Campbell et al., 2018). Its antimicrobial activity and host cytotoxicity are time-dependent with an ability to dissolve necrotic tissue debris (Campbell et al., 2018).

### 6.1 Clinical outcomes

Despite sodium hypochlorite's utilization for centuries as an antiseptic agent, there are limited studies reporting efficacy against biofilm in PJI management. Ernest et al. (2018) compared povidone iodine, hydrogen peroxide, and sodium hypochlorite against MSSA biofilms grown in vitro on cobalt chrome (CoCr), titanium alloy (Ti), and stainless steel (SS) discs. Although all topical applications resulted in reductions in MSSA, the largest bacterial reduction was found in stainless steel implants treated with sodium hypochlorite. Further in vivo studies are needed to evaluate the role of sodium-hypochlorite solution in the treatment of PJI.

## 7 Hypochlorous acid

Hypochlorous acid (HOCl) is a naturally occurring molecule generated by white blood cells during the oxidative burst to kill pathogens (Block and Rowan, 2020). Similar to sodium hypochlorite, the residual chloride ions oxidize the surrounding bacterial or fungal cells in a more acidic pH of 5.5 (Block and Rowan, 2020). HOCl has a focal role in innate host defense with potency against drug-resistant bacteria (Block and Rowan, 2020). HOCl is commercially available as Vashe Wound Therapy Solution (SteadMed Medical LLC, Ft. Worth, Texas) as an irrigation solution marketed for the management of stasis ulcers, diabetic ulcers, and burns (Vashe, 2021).

### 7.1 Clinical outcomes

Commercially available HOCl has demonstrated efficacy against biofilm disruption with low cytotoxic effects (Robson, 2014). Robson (2014) investigated the efficacy of Vashe Wound Therapy Solution against *S. aureus* biofilm and found greater than 99 % reduction following 1 min and 100 % after 3, 5, 7, and 10 min of exposure (Robson, 2014). However, Kubacki and Gilbert (2018) reported HOCl producing significant erosion and wear on cobalt chrome and titanium metals, potentially limiting its utility in DAIR procedures. Further research is needed to determine the utility of HOCl against common PJI pathogens, host tissue toxicity, and its role as an antiseptic irrigant in PJI management.

**Table 2:** Preparation of most common irrigation solutions.

Solution	Additive	Irrigation preparation
Povidone iodine	Antiseptic	17.5 mL 10 % PI + 500 cc NS or Surgiphor (0.5 %) (Surgiphor Wound Irrigation System FDA, 2021)
Chlorhexidine gluconate	Antiseptic	Irrisept (0.05 %) (Premkumar et al., 2020)
Acetic acid	Antiseptic	Available in 3 % concentration without dilution
Sodium hypochlorite	Antiseptic	Dakin's solution (0.5 %) Can be further diluted with 500 cc NS for 0.25 % concentration
Hypochlorous acid	Antiseptic	Vashe Wound Therapy Solution (Vashe, 2021)
0.1 % polyhexamethylene biguanide 0.1 % betaine	Antiseptic–surfactant combination	Prontosan Wound Irrigation Solution (B. Braun, 2021)
Ethanol Acetic acid Sodium acetate Benzalkonium chloride Sterile water	Antiseptic–surfactant combination	Bactisure Wound Lavage solution (Bactisure™, 2021)

PI: povidone iodine; NS: normal saline 0.9 %; L: liter; PA: Pennsylvania; GA: Georgia.

**Table 3:** Antiseptic combination reactions.

Antiseptic solutions	Chlorhexidine gluconate 4 %	Hydrogen peroxide 3 %	Sodium hypochlorite 0.5 %
Povidone iodine 10 %	Precipitate	No reaction	Gas
Hydrogen peroxide 3 %	Precipitate	n/a	Gas
Sodium hypochlorite 0.5 %	Precipitate, gas	Gas	n/a

n/a: not applicable.Table content reproduced with permission from Campbell et al. (2018).

## 8 Preformulated combination irrigant

Bactisure Wound Lavage solution (Next Science Ltd, Jacksonville, Florida; distributed by Zimmer-Biomet, Warsaw, Indiana) is a preformulated irrigation solution that consists of ethanol, acetic acid, sodium acetate, and benzalkonium chloride in sterile water. Bactisure's mixture of surfactants, chelating agents and salts deconstruct the extracellular polymeric substance (EPS) matrix that serves as a physical barrier on bacteria and is fundamental in biofilm formation (Hunter and Duncan, 2019). Prontosan Wound Irrigation Solution (B. Braun Medical Inc, Bethlehem, PA) is a commercially available combination comprised of 0.1 % polyhexamethylene biguanide and 0.1 % betaine (surfactant) that has been reported to have efficacy against biofilms in chronic wound ulcers (B. Braun, 2021); however, its utility in prevention or management of PJI is yet to be determined.

### 8.1 Clinical outcomes

Similar to commercially available HOCl, Bactisure has been primarily studied for the management of skin and soft tissue infections, with inconclusive data on its efficacy in PJI. A prospective, multi-center single-arm study recently concluded that studied the role of Bactisure in TKA PJI and compared preoperative and postoperative aspiration fluid cell counts after articular irrigation with Bactisure in TKA PJI. Since the investigation recently finished, the study's findings and relevancy are unknown. Further studies are needed before a conclusive recommendation can be made on its use in PJI. The preparation of the most common antiseptic irrigation solutions is summarized in Table 2.

**Table 4:** Antiseptic irrigation protocols in the literature.

Study	Antiseptics	Protocol	Success Rate	LOE${}^{a}$
Williams et al. (2017)	AA 3 %	Surgical debridement → modular component exchange → 20 min AA soak → NS irrigation	86.9 % (20/23) TKA at 18 months	Therapeutic Level II
Byren et al. (2009)	CHG${}^{b}$	Surgical debridement → modular component exchange → CHG${}^{b,c}$ pulse lavage	73.1 % (38/51) TKA 86.5 % (45/52) THA at 2.3 years	Therapeutic Level III
Barros et al. (2019)	CHG${}^{b}$	Surgical debridement → 3 L CHG${}^{b}$ irrigation → 3 L NS irrigation → re-drape → 1 L NS irrigation → modular component exchange	89.5 % (34/38) TKA/THA at 3.5 years	Therapeutic Level III
Hart et al. (2019)	PI 0.25 %	Surgical debridement → 1 L PI 0.25 % 3 min soak → NS irrigation	TKA: 96.8 % (487/503) at 3 months 93.4 % (298/319) at 12 months THA: 96.3 % (367/381) at 3 months 94.8 % (219/231) at 12 months	Therapeutic Level III
Kim et al. (2015)	PI 10 %	Surgical debridement → 3–6 L NS pulse lavage → modular component exchange versus head and liner replacement after 10–15 min 97 % ethanol soak → PI 10 % soak 5–10 min → 3 L NS irrigation	100 % THA at 1 year	Therapeutic Level III
Riesgo et al. (2018)	PI 0.35 %	Surgical debridement → modular component exchange → 500 mL PI 0.35 % bulb irrigation → 1 L NS pulse lavage → 1 g Vancomycin deep to fascia, 1 g Vancomycin superficial	TKA: 75 % (12/16) THA: 90 % (8/10) All at 1 year	Therapeutic Level III
George et al. (2015)	PI 10 % H${}_{2}$O${}_{2}$ 1.5 %	Surgical debridement → explantation → 12 L warm NS pulse lavage → 100 mL H${}_{2}$O${}_{2}$ 3 % in 100 mL of sterile water irrigation → NS${}^{c}$ irrigation → 200 mL PI 10 % irrigation → PI 10 % soaked gauze in wound for re-drape → 200 mL PI 10 % irrigation → 1 L NS pulse lavage → component implantation → 1 L NS irrigation	100 % (5/5 THA at 5 years; 28/28 TKA at 6.5 years)	Therapeutic Level II
Haddad et al. (2015)	PI${}^{b}$ H${}_{2}$O${}_{2}^{b}$	Surgical debridement → explantation → PI${}^{b,c}$ and H${}_{2}$O${}_{2}^{b,c}$ irrigations → PI${}^{b,c}$ soak → re-drape → “lavage” → component implantation	One-Stage: 100 % (28/28) at 3 years Two-Stage: 93.2 % (69/74) at 3 years	Therapeutic Level III
Royo et al. (2013)	PI${}^{b}$ H${}_{2}$O${}_{2}^{b}$	Surgical debridement → “9 L NS, PI${}^{b}$, H${}_{2}$O${}_{2}^{b}$ irrigation” → modular component exchange → drain with continuous NS irrigation for 24 h (6 L/d rate)	73.5 % (25/34) TKA at 7 months	Therapeutic Level III
Duque et al. (2017)	Bacitracin${}^{b}$ PI${}^{b}$ SH${}^{b}$	Surgical debridement → modular component explantation → 3 L PI${}^{b}$ irrigation → PI${}^{b}$ scrub brush mechanical scrub → 3 L SH${}^{b}$ irrigation → 3 L Bacitracin${}^{b}$ irrigation → 3 L NS irrigation → re-drape → component implantation	69 % (46/67) TKA 85 % excluding MRSA infections	Therapeutic Level III

${}^{a}$ Marx et al. (2015).${}^{b}$ Concentration not specified.${}^{c}$ Volume not specified.LOE: level of evidence, MRSA: methicillin-resistant staphylococcus aureus, NS: normal saline, PI: povidone iodine, PJI: periprosthetic joint infection,SH: sodium hypochlorite, THA: total hip arthroplasty, TKA: total knee arthroplasty.

**Table 5:** Grades of recommendation for irrigation fluids in the management of periprosthetic joint infection.

Additives	Grade of recommendation*	Recommendation
Surfactants	B	Should not be added to irrigation
Antibiotics	A	Should not be added to irrigation
Antiseptics	C	May be added, but studies are too mixed to determine an optimal antiseptic

* According to Wright (2006), grade A indicates good evidence (Level I studies with consistent findings) for or against recommending intervention; grade B, fair evidence (Level II or III studies with consistent findings) for or against recommending intervention; grade C, poor-quality evidence (Level IV or V studies with consistent findings) for or against recommending intervention; and grade I, insufficient or conflicting evidence not allowing a recommendation for or against intervention.Table content reproduced with permission from Kavolus et al. (2020).

## 9 Complications of antiseptic solution combinations

Although the combination of antiseptic solutions has demonstrated synergistic bactericidal effects and broader antibacterial coverage, it is important to note that not all irrigants should be used concomitantly; 1 % povidone iodine, 0.25 % acetic acid, 3 % hydrogen peroxide, and 0.5 % sodium hypochlorite have demonstrated substantial host cytotoxicity, especially when used together (Lineaweaver et al., 1985). A 10 % povidone-iodine solution combined with 4 % CHG has been reported to result in precipitates. Similarly, a 10 % povidone-iodine solution combined with 7.5 cc of 0.5 % sodium hypochlorite has also resulted in both a precipitate and gas product. While the combination of hydrogen peroxide and povidone iodine does not form a precipitate or gas, a 3 % hydrogen-peroxide solution mixture with 4 % CHG forms precipitates and potentially harmful gas when mixed with 0.5 % sodium hypochlorite (Campbell et al., 2018). The effect of precipitates and gas products, however, has yet to be determined.

Particular care must be heeded when combining CHG with other antiseptics, as it can form a precipitate with sodium hypochlorite, 3 % hydrogen peroxide, and 10 % povidone iodine (Campbell et al., 2018). It also forms a potentially flammable and carcinogenic gaseous byproduct when combined with sodium hypochlorite (Campbell et al., 2018). Surgeons should exercise caution while using multiple irrigating solutions and consider adding meticulous irrigation with normal saline between the use of varying antiseptic irrigation solutions (Table 3). Finally, it should be noted that although multiple irrigants are utilized for PJI, the current available antiseptic solutions are not FDA-approved, aside from Bactisure, to be used internally and are reserved for external use only.

## 10 Conclusion

This general overview provides the most up-to-date review of the available literature on irrigation solutions used in PJI. While basic science studies assist in the selection of irrigation fluids, in vitro results do not directly translate into clinical significance once implemented in vivo. Direct comparison of different irrigation solutions remains difficult because of variability in treatment protocols (Table 4). The current commercially available antiseptic irrigants all have potential against a broad spectrum of PJI pathogens with their own unique advantages and disadvantages (Table 5). PI, CHG, hydrogen peroxide, acetic acid, Dakin's solution are all cost-effective options with noteworthy in vitro bactericidal properties. It is important to consider that although solutions used in combinations demonstrate excellent efficacy against common pathogens in vitro, there is inconclusive data on in vivo efficacy and complications related to the observed byproducts. It is also important to be aware that many of the current available antiseptic solutions are not FDA-approved for internal use. Commercially preformed irrigants are FDA-approved wound lavage solutions that have shown early promise; however, the increased cost and role in the management of definitive PJI treatment is yet to be determined. Therefore, there are currently no clear recommendations regarding the optimal irrigation solution for management of acute or chronic infections. Future clinical studies are needed to identify the ideal irrigation solution(s) with optimal bactericidal properties and low cytotoxicity for PJI treatment.

## Data Availability

No data sets were used in this article.
